# Strain induced polarization chaos in a solitary VCSEL

**DOI:** 10.1038/s41598-017-14436-3

**Published:** 2017-10-25

**Authors:** T. R. Raddo, K. Panajotov, B.-H. V. Borges, M. Virte

**Affiliations:** 1 Brussels Photonics Team (B-PHOT), Department of Applied Physics and Photonics (TONA), Vrije Universiteit Brussel, Pleinlaan 2, 1050 Brussels, Belgium; 2 Department of Electrical and Computer Engineering, EESC, University of São Paulo, 13560-250 São Carlos-SP, Brazil; 3 Institute of Solid State Physics, 72 Tzarigrasko Chausse Blvd., 1784 Sofia, Bulgaria

## Abstract

Physical curiosity at the beginning, optical chaos is now attracting increasing interest in various technological areas such as detection and ranging or secure communications, to name but a few. However, the complexity of optical chaos generators still significantly hinders their development. In this context, the generation of chaotic polarization fluctuations in a single laser diode has proven to be a significant step forward, despite being observed solely for quantum-dot vertical-cavity surface-emitting lasers (VCSELs). Here, we demonstrate experimentally that a similar polarization dynamics can be consistently obtained in quantum-well VCSELs. Indeed, by introducing anisotropic strain in the laser cavity, we successfully triggered the desired chaotic dynamics. The simplicity of the proposed approach, based on low-cost and easily available components including off-the-shelf VCSELs, paves the way to the wide spread use of solitary VCSELs for chaos-based applications.

## Introduction

## Polarization chaos from a solitary VCSEL

Besides its interest as a test-bed for a better understanding of the behaviour of complex nonlinear dynamical systems, optical chaos has been smartly exploited in chaotic laser radar^1^, secure communication or key distribution^2–6^, and high-speed random bit generators^7–10^. For all these applications, a laser subject to optical injection and/or time-delayed feedback is typically used as a source of chaos^11,12^. But this solution is also relatively complex to setup, run and maintain. In addition, it also makes the whole system quite expensive, hence creating a major hurdle that limits both the attractivity and further developments of these schemes. The discovery of polarization chaos^13–16^, i.e. chaotic polarization fluctuations that can be generated directly from a solitary laser diode, was expected to significantly change the current scenario. Indeed, having a single tiny laser generating chaotic fluctuations without any external perturbation would be the simplest and most efficient way of generating optical chaos. Nevertheless, polarization chaos has only been observed so far in one specific type of semiconductor laser: the quantum dot vertical-cavity surface-emitting lasers (VCSELs), grown and described by Hopfer *et al*.^17^. Even though the observation of polarization chaos has proven to be a very promising result, the commercial non-availability of such devices makes this optical source disconnected from real-world applications.

In this article, we overcome this hurdle and demonstrate polarization chaos dynamics in a commercially available quantum-well (QW) VCSEL on which we mechanically applied in-plane anisotropic strain. By changing the orientation and the amount of applied strain, we tune the internal parameters of the gain medium^18–22^ to successfully generate the desired chaotic output. Despite the emergence of a second-order mode and the difference in its polarization, our observations are in excellent agreement with previous experimental results reported for QD VCSELs^15^. Moreover, we also unambiguously confirm the deterministic nature of the dynamics via numerical processing of experimental data.

As a result, the impact of our work is two-fold: (1) it confirms that the polarization chaos dynamics – observed in ref.^15^ – was not linked with the quantum dots used as gain medium, and therefore demonstrates that the dynamics originates from the VCSEL structure itself. (2) it shows that potentially all VCSEL devices could be turned into optical chaos generators if the appropriate strain level is applied.

## Triggering polarization chaos through anisotropic strain

**Figure 1:**
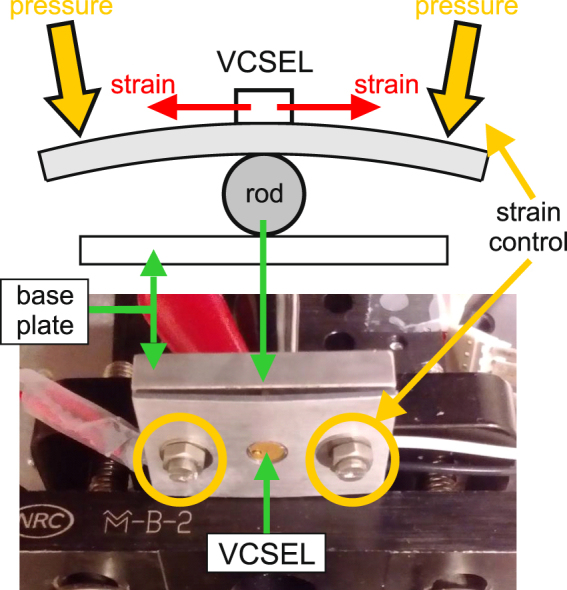
Custom-made holder for strain inducing technique. Two screws are used to control the level of strain. A thermistor and a Peltier element have been added for temperature control and stabilization.

We use here a commercial QW VCSEL (ULM850-PM-TN-S46XZP from Philips Photonics) in a TO46 package emitting at 850 nm with a lasing threshold around 0.5 mA and stabilized at a temperature of 22 °C. The cap of the TO package of the QW VCSEL is removed so that the mechanical stress can be applied. A steel rod placed between a metal base plate and a pressing cover plate as seen in Fig. 1 is used to bend the packaged VCSEL, see further details in section A of the Supplementary Information. Although this technique has the clear advantage of simplicity, its main drawback is that it does not allow the level of applied strain to be measured unlike other more complex approaches^23,24^. Nevertheless, this technique is sufficient to change the gain medium anisotropies as experimentally confirmed in ref.^18^. Going a bit further, an anisotropic stress applied onto a quantum well gain medium induces polarization dependent variations of both the gain and the refractive index^19–21^, which in turn directly impacts the nonlinear polarization dynamics of the laser itself^22^. Although this approach could lead to a more complex system of equations as those used in ref.^15^, we are at this point only interested in successfully triggering polarization chaos experimentally using this technique.

The emitted laser beam is collimated by an aspherical lens and is coupled afterwards into a multimode fibre. An optical isolator is used to avoid unintentional back reflections from the fibre front-facet A half-wave plate is put in front of the oscillator to select the polarization direction to be analysed. We define the 0° reference as the direction of the linearly polarized (LP) light emitted at threshold when no stress is applied. Thus, the two preferred polarization axes, the so-called laser polarization eigenmodes, correspond to the polarization at 0° and 90°, respectively.

We carry out both static and dynamic polarization-resolved measurements. For the former, we use a power meter and an optical spectrum analyser (ANDO AQ6317), while for the latter we exploit a high-speed photodetector (NewFocus 1554-B, 12 GHz) followed by an electronic amplifier, combined with an electrical spectrum analyser (Anritsu, MS2667C, 30 GHz) or an oscilloscope (Tektronix CSA7404, 4 GHz, 20 GSamples/s).

**Figure 2:**
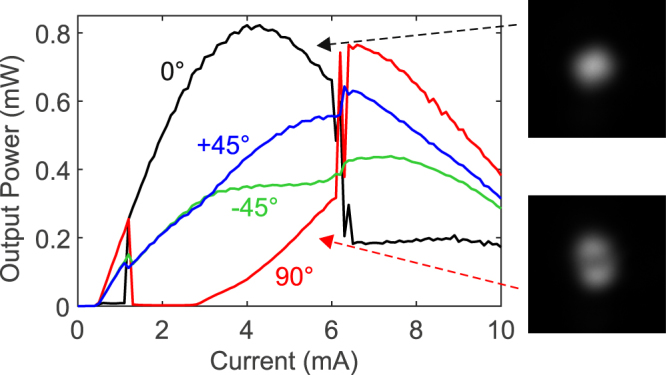
Polarization resolved output power for increasing injection current. A relatively high level of strain is applied to the device and polarization chaos is observed for injection currents above 6.2 mA. The two insets show the spatial profile for polarization at 0 and 90° for 6 mA injection current - just before the second switching -, which confirms the existence of a 2^nd^ order mode at high current levels (bottom right inset).

The impact of the strain on the polarization behaviour strongly depends on its orientation. In our case, it appears to be more efficient to apply the strain orthogonally to the polarization direction at threshold without strain, i.e. with the steel rod placed along the 0° axis. Theoretically, polarization chaos has been linked with polarization switching (PS) events of type II^16^, i.e. from the low to high-frequency eigenmode, as opposed to a switching from high to low frequency eigenmode which is type I. Even though we were not able to measure the frequency splitting between the two polarization eigenmodes due to the limited resolution of the OSA - and therefore not able to discriminate between type I and type II switching -, we looked for PS events as a first indicator to identify potential cases of interest. By gradually increasing the amount of strain, we destabilized the laser and successfully obtained a double PS scenario, as shown in Fig. 2. At this strain level, the laser starts emitting from the orthogonal eigenmode (90°) at threshold. When increasing the injection current, the first PS occurs around 1.3 mA: the measured power at 90° goes extinct whereas the power at 0° increases suddenly. Then, the second PS occurs around 6.2 mA.

**Figure 3:**
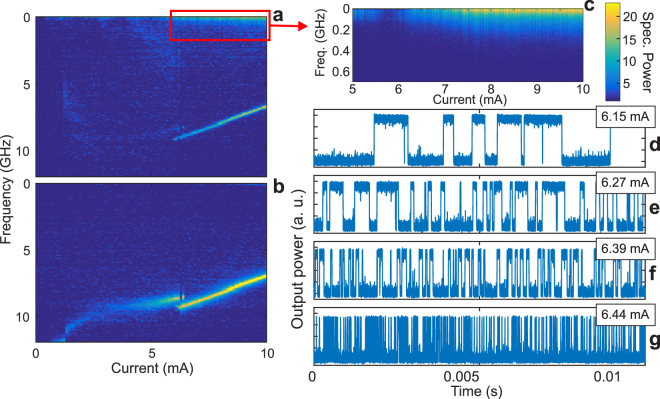
Dynamical measurements. Evolution of the Radio Frequency spectrum for increasing injection currents for polarization at 0° (**a**) and at 45° (**b**). Panel (c) shows the zoomed-in plot highlighted by the red rectangle in (**a**) showing the featureless low-frequency contribution corresponding to the mode-hopping dynamics. The same colormap is used for (**a**,**b**,**c**) and shows the spectral power in dB/Hz normalized by the noise floor measured with no optical signal. In (**d**–**g**), we show time-series recorded for polarization at 90° showing random-like hopping between polarization modes with a significant decrease of residence time for increasing injection current at: 6.15, 6.27, 6.39 and 6.44 mA respectively.

Double switching has only been reported in the literature as a PS of type I followed by a PS of type II^25,26^. Thus, we can reasonably assume that the second PS is of type II, and therefore that a complex dynamical behaviour might emerge. Consequently, we perform dynamical measurements focusing on the current range around the second PS event. The corresponding results are shown in Fig. 3. Panels a and b show the evolution of the radio frequency (RF) spectra recorded for the polarization at 0 and 45° when increasing the injection current. At large currents, only a single peak appears for the polarization at 45° with no other visible frequency component, hence suggesting a behaviour close to a periodic solution with a frequency around 8 GHz. A sudden frequency change of about 600 MHz appears at the onset of the second switching which clearly reminds the limit cycle bistability reported in chaotic QD VCSELs^27^. In contrast, although the same peaks can be observed for the polarization at 0° as displayed on panel a, we also observe the emergence of a featureless part in the low frequency range (below 1 GHz) as highlighted in the zoomed-in plot of panel c. This suggests that a relatively slow dynamical behaviour emerges for higher currents on top of the oscillations around 8 GHz. The lack of any features indicates that the corresponding dynamical behaviour does not exhibit any characteristic frequency, which suggests a chaotic behaviour. Based on recorded time-series for polarization at 0°, we confirm the observation of a random-like hopping dynamics, see panels (d),(e),(f) and (g) of Fig. 3. The evolution of the residence time for increasing currents remains however similar as it exhibits an exponential decrease as the injection current is increased, as opposed to the evolution observed for stochastic mode hopping^28^: this trend can be confirmed in Fig. 3 and additional statistical details are given in section B of the Supplementary Information. These features are in excellent agreement with previous observations of polarization chaos^15^. We should however point out that, while polarization chaos in QD VCSELs shows an evolution of the residence time from seconds to nanoseconds when varying the injection current^14^, the shortest average residence time recorded for the strained QW VCSELs lies in the microsecond range. Although the reason for this limitation remains unclear at this stage, considering the excellent qualitative agreement obtained with polarization chaos features, we are convinced that these dynamics have the same origin.

Finally, to fully confirm the deterministic nature of the dynamics, we use the same tests as detailed in ref.^15^. Using Wolf’s algorithm, we confirm that the dynamics exhibits a non-zero but finite value of the largest Lyapunov exponent in line with our chaotic interpretation. The Grassberger Procaccia algorithm yields a non-zero but finite estimate of the *K*_2_-entropy (*K*_2_ = 5.2 10^−3^ ns^−1^) with a corresponding correlation dimension *D*_2_ ≈ 2.04. Although the correlation dimension is very close to the value reported previously, the *K*_2_-entropy is significantly lower than the one reported in^15^, which can be explained by a much slower hopping dynamics. Since the critical point here is to have a finite *K*_2_-entropy, both results confirm that the reported dynamics is indeed fully deterministic. Further details on these tests and their results can be found in section C of the Supplementary Information. Overall this battery of test confirms the chaotic nature of the reported behaviour and therefore rules out stochastic processes as the physical origin of the phenomenon.

## Peculiarities of the observed dynamics

The dynamical measurements confirm that we have indeed triggered chaotic polarization fluctuations. However, the observed behaviour also differs from that of QD VCSEL in some aspects.

**Figure 4:**
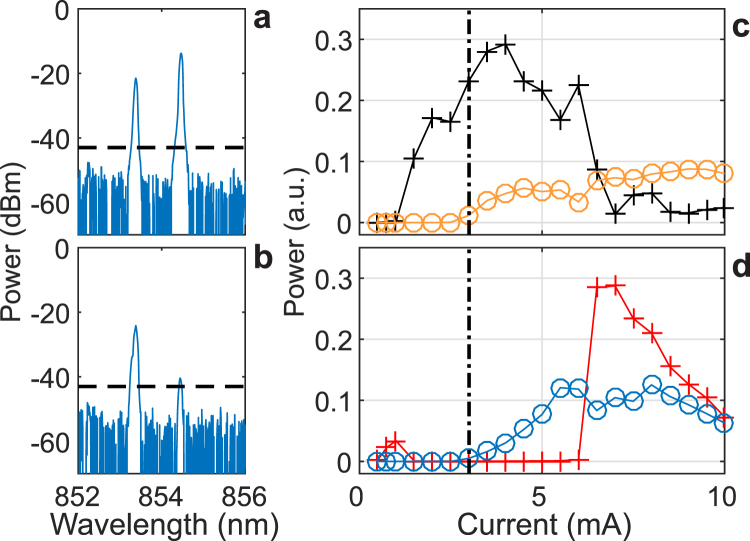
Frequency resolved LI-curves. (**a**,**b**) Typical two-mode optical spectra recorded at 4 mA for polarization at 0° (**a**) and 90° (**b**). The horizontal dashed lines indicate the threshold above which spectral data are integrated to estimate the power of each mode. (**c**,**d**) Frequency resolved LI curves for polarization at 0° (**c**) and 90° (**d**): the black (red) solid line with crosses represents the fundamental mode while the orange (blue) solid line with circles represents the second order mode. The vertical dash-dotted line indicates the threshold for the second order mode, i.e. about 3 mA.

First, the laser diode does not emit only a single spatial mode: the manufacturer recommends a maximum injection current of 3 mA, i.e. about 6 times the laser threshold. This recommendation aims not only at extending the device lifetime, but also at preventing the onset of higher-order spatial modes. Above this limit, the laser emits a second-order transverse mode, which is confirmed by the typical two lobes spatial profile displayed in Fig. 2. This mode is expected to emerge with a polarization orthogonal to that of the fundamental mode^29^. Therefore, the increasing power for the polarization at 90°, for currents above 3 mA, cannot be directly interpreted as a transition through an elliptically polarized (EP) mode, which has been reported before the onset of polarization chaos^15,16^. As a result, we need to make sure that the observed random-like hopping between polarization states is not a switching between spatial modes. To this end, we exploit the wavelength difference between the two spatial modes (>1 nm, see Fig. 4 (a,b)) to record frequency-resolved light-intensity (LI) curves for polarization at 0° and 90°. We measure optical spectra at different current levels, and estimate the output power for each spatial mode by integrating the spectral contribution of the corresponding peak. We set a threshold at −42 dBm below which we consider that there is only noise and no signal. These LI curves are given in Fig. 4(c) and (d), for polarization at 0° and 90° respectively. Similar LI curves for polarization at 45° and −45° are also available in section D of the Supplementary Information. We observe that, for both polarization orientations, the contribution of the second order mode remains roughly constant despite the PS event. However, a large and sudden change is observed in the polarization of the fundamental mode. This confirms that the second switching is mainly due to the fundamental mode itself. Moreover, when zooming around the switching point, we also notice that the fundamental mode undergoes a short transition through a slightly elliptical polarization just before the second switching point, around 6.2 mA (see the inset of panel (b) in Figure 10 of the Supplementary Information). This is important, as this transition has been shown to be an essential step in the route to polarization chaos^16^. We found similar results when spatially filtering out the second order mode with an iris diaphragm after expansion of the laser beam.

**Figure 5:**
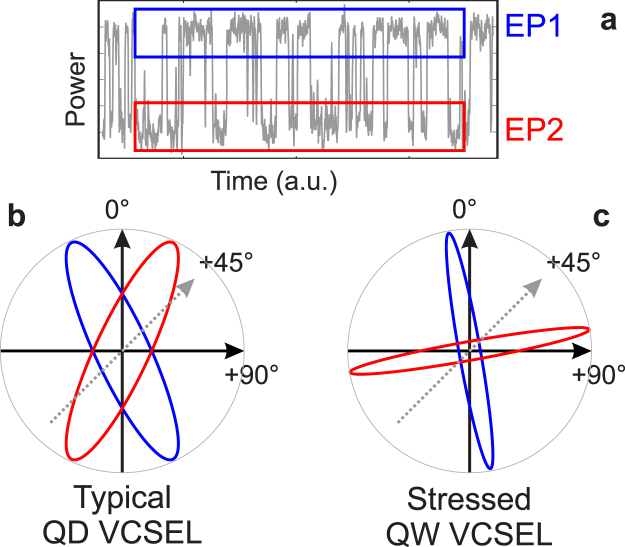
Orientation of the elliptically polarized states. (**a**) Typical polarization chaos time-series showing a random-like hopping between two elliptically polarized states EP1 and EP2 highlighted in blue and red, respectively. The orientation of these two states is shown with respect to the laser eigenmodes for a typical case in QD VCSEL and for the stressed VCSEL described here in (**b**) and (**c**), respectively.

Second, the chaotic dynamics resembles a random-like hopping between two EP states, as shown in Fig. 3 and highlighted in Fig. 5(a). These are obtained after the fast oscillations around 8 GHz, corresponding to the rotation around the scrolls of the chaotic attractor, have been filtered out^30^. In QD VCSELs, these two EP states have a strong ellipticity and are typically symmetrical with respect to the laser eigenmodes (i.e. the 0° and 90° axes), as schematically illustrated in Fig. 5(b). Small asymmetries between EP state 1 (EP1) and EP state 2 (EP2) have already been reported, but were deduced either from asymmetries in the residence time statistics^31^ or from the occurrence of dynamical bistability^27^. Indeed, no significant asymmetries could be spotted between the polarization orientation of the two EP states. Consequently, the largest amplitude for the random-like hopping was always obtained for a projection at 45° of the polarization direction at threshold^14^.

In contrast, stressed QW VCSELs present a different behaviour as illustrated in Fig. 5(c). The two EP states are very close to the laser eigenmodes themselves and therefore show no sign of symmetry with respect to the 0° and 90° axes. As a result, the random-like hopping is observed for projection at 0° or 90° and not at 45°, unlike what has been previously reported^27,31^. Moreover, the ellipticity of the two states is much smaller than that of the EP states in QD VCSELs. Yet, the asymmetries in the residence time statistics - shown in Supplementary Information - and the frequency difference between the periodic solutions oscillating around the two EP states - about 600 MHz, as shown in Fig. 3 - are of the same order of magnitude as those reported in^27,31^. The difference might therefore be limited to only the EP state orientation and ellipticity with little impact on the other dynamic properties.

## Discussion and Perspectives

Even though the emergence of a higher-order mode and the differences in the polarization of the modes are an undeniable proof of the higher complexity of the system, our observations confirm that we successfully generated polarization chaos in a solitary quantum well VCSEL. We have achieved it using only commercially available QW VCSELs subject to mechanical strain without any other external forcing. Polarization chaos consistently appeared for the handful of devices from Philips Photonics that we have tested with a good repeatability. The simplicity of the strain technique guarantees that the proposed optical chaos generator can be reproduced easily, and combined with already low-cost VCSELs. Although the appearance of a higher-order mode made the situation more complex, we were still able to confirm that the reported dynamics is identical to the chaotic behaviour presented in ref.^15^. Besides an excellent agreement with experimental observations, we also unambiguously confirmed the chaotic nature of the dynamics via numerical processing of experimental time-series. For simplicity, a list of all the essential features confirming the chaotic dynamics has been compiled in section E of the Supplementary Information. Hence, this work solved for the first time the practical problem of unavailability of devices capable of generating polarization chaos^15^ and reveals that all VCSEL devices could potentially be turned into chaos generators. From a more fundamental point of view, this result also confirms that the chaotic dynamics originates from the VCSEL structure itself and is not related to the quantum dots as gain medium as it could have been mistakenly foreseen from previous observations.

Finally, this optical chaos generator might significantly influence emerging applications that are currently impractical or infeasible due to complexity, high cost, and limited availability of known solutions. We strongly believe we have paved the way for future research on polarization chaos, its properties and related applications that can largely benefit from the simplicity and compactness of the proposed approach. In addition, the absence of time-delay (such as in schemes based on optical feedback) entirely removes the need for difficult time-delay concealment^32–34^. The present work also motivates further studies on strained VCSELs capable of generating chaotic polarization fluctuations without any external mechanical forcing, e.g. using integrated thermal tuning^35^. This possibility opens the way towards advanced chaotic laser systems and applications, such as two-dimensional arrays of chaotic devices comprising hundreds of individual sources which could be highly desirable e.g. for massive on-chip parallelization of polarization chaos-based random bit generators^30^. Besides, following the recent progress using alternative strain-inducing techniques, where self-pulsations at more than tens of GHz and beyond have been demonstrated^23,24^, the proposed scheme might also have the unique potential to achieve chaotic dynamics at extremely high frequencies, which would certainly represent a major paradigm-shift in the design of optical chaos-based systems.

## Methods

### Polarization resolved Light-Intensity curve

The polarization direction chosen for analysis is manually selected via the half-waveplate placed before the optical isolator. Thus, the LI curves for different polarizations are obtained sequentially and for increasing injection current only.

### Radio-Frequency map

With the same setup, the polarization resolved light output is sent to a fast photodetector (NewFocus 1554-B, 12 GHz) connected to a Radio-Frequency Spectrum Analyzer (Anritsu, MS2667C, 30 GHz). The injection current is increased step by step. For each step, we implement a pause of 10 seconds before acquiring the RF spectrum to let enough time for the scanning process to be completed. As for the LI curves, the maps for different polarization orientations are acquired sequentially. Finally, all data are normalized by the averaged (5 takes) spectrum measured when no optical signal is present, i.e. when the laser is switched off, so that only variations due to the optical signal appear on the map. We scan frequencies in the range from 0 to 12 GHz, except for the zoom where additional measurements have been performed with a scanning range from 0 to 1 GHz.

### Polarization and frequency resolved light-intensity curves

With the same setup, the polarization resolved light output is sent to an Optical Spectrum Analyzer (OSA) (ANDO AQ6317). The injection current is increased step by step, and for each step the optical spectrum of the laser beam is acquired. Then, for all spectra, we apply a threshold at −42 dBm and identify the peaks in the resulting output. The relative position of the peaks is then used to identify the fundamental mode (larger wavelength) and other spatial modes (shorter wavelength), and the total power for each mode is then obtain by integration over all the points around the peak and above the threshold.

### Far-Field profile and spatial mode filtering

To obtain the far-field profile of the laser beam, we extend the previous setup. We spatially expand the laser beam with a pair of lenses and use a CCD camera (Grey Point, Chameleon, monochrome) for the image acquisition to filter out the second order spatial modes, we place an almost closed iris after the beam expansion to only keep the central part of the far-field image which corresponds to the fundamental mode.

The datasets generated during and/or analysed during the current study are available from the corresponding author on reasonable request.

## Electronic supplementary material

Supplementary information
