# Supplementary material for: Strain induced polarization chaos in a solitary VCSEL
Source: Sci Rep. 2017 Oct 25;7:14032. doi: 10.1038/s41598-017-14436-3 (PMC5656599; doi:10.1038/s41598-017-14436-3)
Supplement: Supplementary file 1 — Supplementary information [file 41598_2017_14436_MOESM1_ESM.pdf]

Strain induced polarization chaos in a solitary VCSEL *(Supplementary Information)*

T. R. Raddo[1, 2], K. Panajotov[1, 3], B.-H. V. Borges[2], and M. Virte[1, †]

[1]Vrije Universiteit Brussel, Department of Applied Physics and Photonics, Brussels Photonics (B-PHOT), Pleinlaan 2, B-1050 Brussels, Belgium
[2]Department of Electrical and Computer Engineering, EESC, University of São Paulo, 13560-250 São Carlos-SP, Brazil
[3]Institute of Solid State Physics, 72 Tzarigrasko Chausse Blvd., 1784 Sofia, Bulgaria
†mvirte@b-phot.org

A. VCSEL holder construction details

In this section, we describe the custom VCSEL holder used to apply anisotropic strain on packaged VCSELs. This holder is similar to the one used in [1] and has been designed for VCSELs in TO46 packages and might need to be adjusted for other packaging.

The holder comprises a main metal plate and a lid. Typical dimensions are displayed in Figure 1. Two screws are used to fix the metal lid onto the VCSEL. Then, by placing a small metal rod behind the VCSEL, we can induce anisotropic strain and tune the level of applied strain by fastening or loosening the screws that fix the metal lid. Finally, a thermistor is placed inside the metal plate and a Peltier element glued on the back of the plate to control the temperature of the system.

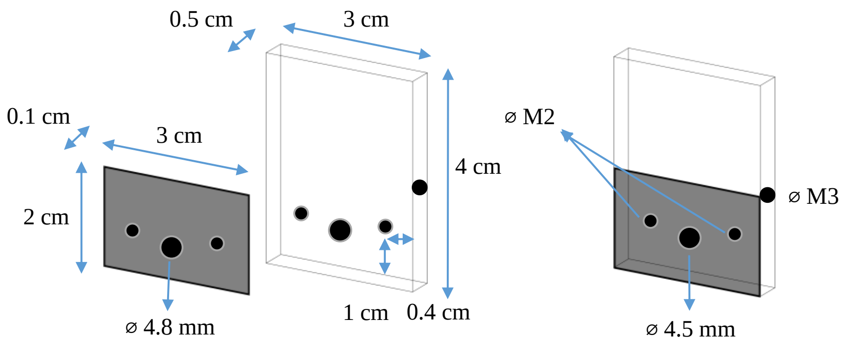

***Figure 1: Schematic dimensions of the VCSEL holder**. The holder consists of a metal plate and a metal lid. The packaged VCSEL is placed behind the central hole of the lid and a steal rod - not pictured - is placed between the VCSEL and the metal plate. Pressure can then be applied on the metal lid and adjusted using the M2 screws - not pictured - on each side of the device. A thermistor is placed into the metal plate's right side hole to control the device temperature.*

B. Polarization chaos statistics: residence time estimate

In Figure 2 , we give a rough estimate of the average residence time - also called dwell-time - for the polarization chaos dynamics obtained in a stressed QW VCSEL. The dataset is relatively small, and therefore we limit our analysis to the apparent trend keeping in mind that the accuracy of the result is not sufficient for a detailed analysis as done in [2].

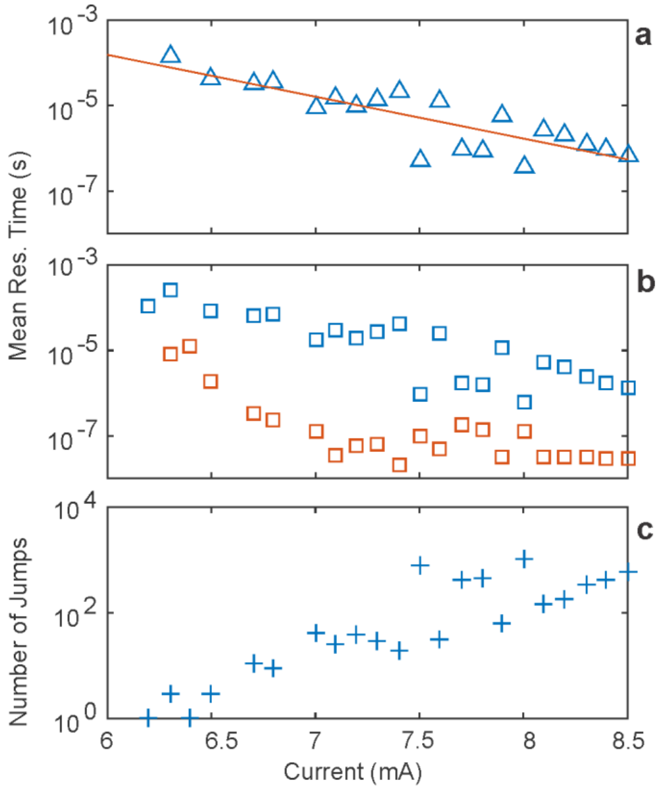

Figure 2: Mode hopping statistics*. (a) average residence time when considering all jumps. The red line gives the following linear fit: -0.98*current + 2.074. (b) Average residence time for the upper (orange) and lower (blue) levels. The upper and lower levels are defined for a time-series recorded at 0° polarization. (c) Number of jumps considered for each point.*

The global trend for the average dwell time is quite clear as shown in panel (a), where a good fit is obtained with a linear approximation. This outcome confirms the exponential decrease of the residence time as the current is increased.

The results shown in (b) are obtained considering separately the lower and upper levels. We can clearly observe a strong gap between the two set of data as a difference of about 2 orders of magnitude is recorded between the residence times of the two levels. Strong fluctuations are also observed but, again, the trend is clear and suggests an asymmetrical behavior as analyzed in [2].

Finally, in the last plot we show the number of jumps versus injection current, which is the number of jumps in the recorded time-series. For low current values, the number of jumps is small but for current levels above 7.5 mA, hundreds of jumps per point have been recorded.

C. Additional time-series processing

1- *Estimation of the Largest Lyapunov Exponent*

Similarly to what has been done in [3], we used the so-called Wolf's algorithm [4] to estimate the largest Lyapunov exponent (LLE) from experimental time-series. The LLE characterizes how fast two nearby trajectories diverge in the system phase space, thus from a theoretical point of view: chaotic systems will exhibit a finite positive (non-zero) LLE while a purely stochastic process will have an infinite LLE and a stable process will have a negative LLE.

In the figure below, we give the evolution of the estimated LLE when increasing the injection current. Although very low LLEs are obtained at low current values, we observe a clear increase at higher injection currents. As discussed in previous work [5], the complexity of the dynamics mostly arises from the jumps between the two scrolls of the chaotic attractor. Thus, we observe a clear correlation between the estimated LLE value and the average residence time as discussed in the next section of the supplementary information.

Overall, the use of Wolf's algorithm clearly yields a finite non-zero value of the Largest Lyapunov exponent coherent with the chaotic interpretation of the dynamics.

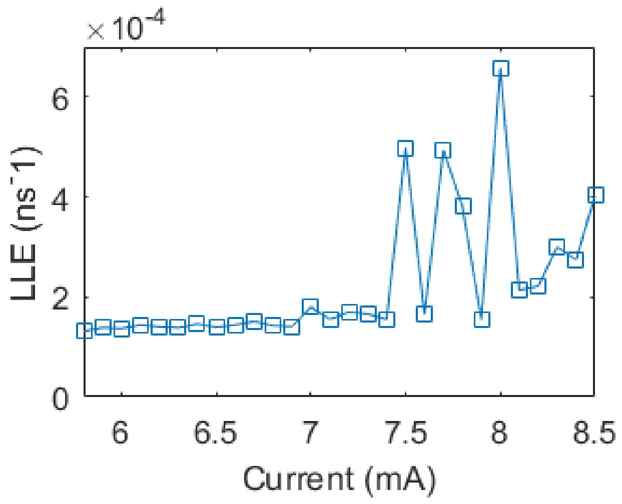

Figure 3: Estimation of the Largest Lyapunov Exponent for increasing injection current using Wolf's algorithm.

2- *Hidden Markov Processes*

Another statistic-based approach to discriminate a deterministic mode-hopping from a stochastic mode hopping consists in modelling the hopping dynamics as Hidden Markov Processes (HMP). Whereas a deterministic dynamics can be modelled without hidden processes, the modeling of a stochastic process does require the inclusion of hidden processes [3,6].

In practice, we use the Baum and Welch algorithm to estimate the corresponding 2x2 transmission and emission matrices of the model, that we will identify as A and B respectively. In our case, we focus on the anti-diagonal terms of matrix B: values close to 0 indicate that no hidden processes appear while non-zero terms indicate otherwise.

When using this approach on the recorded time-series, the terms of the anti-diagonal of matrix B appear to be close to 0: typically, well below 10^{-4} and always below 10^{-2}. These results therefore indicate that no hidden processes are required to accurately model the mode-hopping dynamics as a two-level Markov process, unlike what would be expected for a noise-induced dynamic.

3- *Grassberger Procaccia Algorithm*

The Grassberger Procaccia (GP) Algorithm is typically used to estimate the so-called K_2 or Kolmogorov entropy from time-series data, and in case the value of the K_2-entropy converges, the algorithm provides an estimate of the correlation dimension of the chaos investigated [7,8]. We use the same approach and same notations as those described in [3], including in particular the re-embedding procedure introduced in [9] before processing the experimental data with the GP algorithm. As can be seen in Figure 4, we obtain a result that is very similar to [3]: the K_2-entropy converges along with the correlation dimension D_2. Based on these results, we obtain a K_2-entropy about K_2 = 5.2 10^{-3} ns^{-1} with the corresponding correlation dimension $D_2 \approx 2.04$. As already briefly discussed in the main text, the correlation dimension is close to the one reported in [3] and the Kolmogorov entropy is strictly positive, which confirms the chaotic nature of the dynamics.

However, the value of the K_2-entropy is three orders of magnitude smaller than the one reported previously, but this can be easily explained considering the different time-scales of the dynamics. In [3] the average dwell-time is of the order of the nanosecond while in this report we only reach the microsecond scale, we therefore use a different sampling rate for the two cases. The delay constant τ used to the computation of the K_2-entropy is therefore three orders of magnitude larger [3,7], hence leading to a significantly smaller value for the slower dynamics.

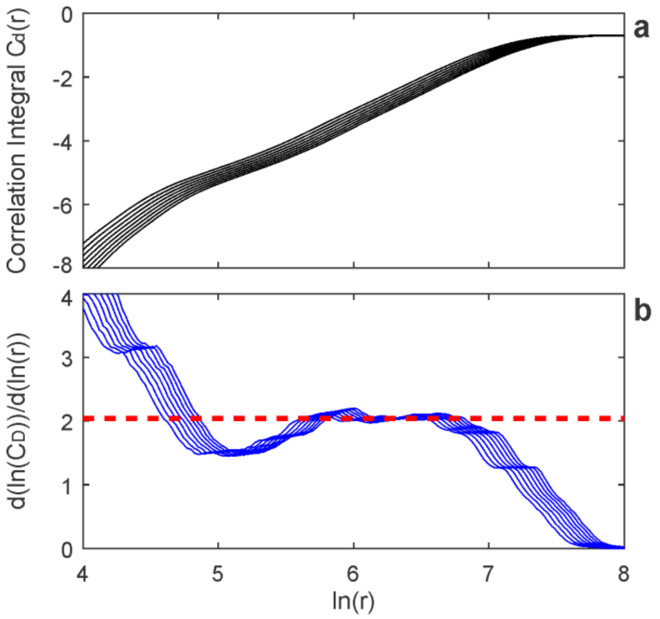

Figure 4: Results of the GP algorithm*. In both panels, we show the evolution of the curves for increasing segment length D. Panel (a) shows the evolution of the correlation integral C_D - i.e. the average number of neighbouring points in the sphere of radius r. In panel (b) we give the derivative $d(ln(C_D))/d(ln(r))$ that leads to the correlation dimension when it converges when increasing the segment length D. Such convergence is clearly obtained around ln(r) = 6.3 where the curves form a clear plateau giving the correlation dimension around 2.04.*

D. Polarization and frequency-resolved optical spectra

In this section, we provide additional details on the emergence of higher-order modes and their polarization.

In Figure 5, we show the frequency resolved LI curves for four different projections at 0, 90, 45 and -45 ° respectively. We use the same convention as described in the text. As already mention, the second switching which appears around 6.2 mA, is clearly a switching of the fundamental mode: while we see a large exchange between polarization at 0 and 90° for the fundamental mode, the second order mode sees only very little changes. In addition, we observe that the projections at 45 and -45° for the fundamental modes are almost identical which confirms that the fundamental mode is almost linearly polarized until the second switching, i.e. below 6.2 mA. Nevertheless, looking closer around the switching point, we see a short transition through a slightly elliptical polarization as shown in the inset of panel (b) in which we observe a small increase for the polarization at 90° just before the switching point.

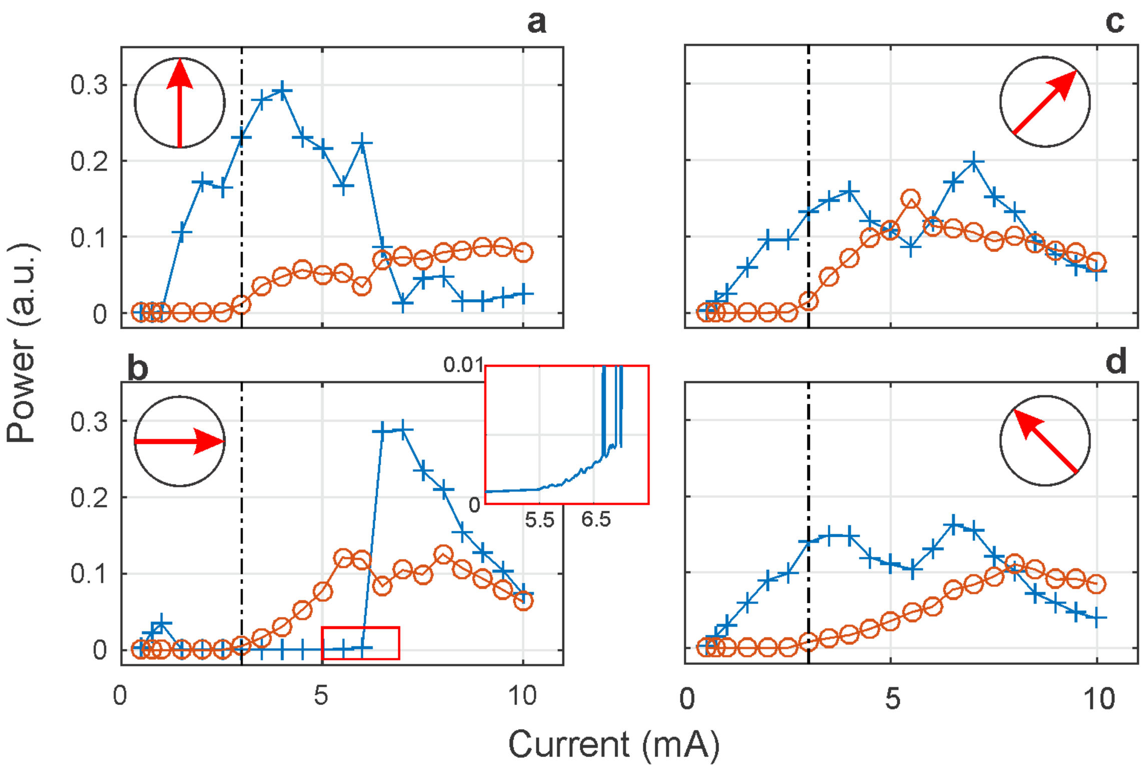

Figure 5: Frequency resolved LI curves for projection at 0° (a), 90° (b), 45° (c) and -45° (d). *For each panel the projection orientation is also indicated by the red arrow in the black circle. In all panels, the blue line with crosses shows the evolution of the fundamental mode while the orange line with circles represent the second order mode. The inset in panel (b) shows a zoom around the switching point delimited by the red rectangle.*

E. List of features confirming the chaotic dynamics

In summary, the dynamics observed from off-the-shelf VCSELs subjected to mechanical strains proved to show the following features:

1. Dynamics following a polarization switching event of type II. We observed double PS event which is typically a type I switching followed by type II.
2. An abrupt frequency shift and a second PS event appear simultaneously. This shift of frequency is similar to the bistability limit cycle observed in chaotic QD-VCSELs [10].
3. The dynamics appears as a random-like hopping between two polarization modes, and the average residence time decreases exponentially for increasing currents.
4. Using Wolf's algorithm, we obtain a positive largest Lyapunov exponent for the dynamics.
5. Modelling the dynamics as a Markov process confirms that no hidden processes are required to obtain and accurate modelling.
6. The Grassberger-Procaccia algorithm converges to a non-zero value of the K_2-entropy (5.2 10-3 ns^{-1}), and a corresponding correlation dimension D_2 equals to 2.01.
7. Frequency resolved measurements confirm that the results reported are mostly due to the evolution of the fundamental mode despite the emergence of a second order mode.
   Using frequency resolved measurements, we observe a short transition through elliptically polarized states consistent with the route to polarization chaos.

All these features are in excellent agreement with theoretical models and previous observations of polarization chaos in quantum dot VCSELs, hence allowing us to conclude that the observed dynamics is indeed polarization chaos.

References:

1 Panajotov K, Nagler B, Verschaffelt G, Georgievski A, Thienpont H, Danckaert J *et al.* Impact of in-plane anisotropic strain on the polarization behavior of vertical-cavity surface-emitting lasers. *Appl Phys Lett* 2000; **77**: 1590.

2 Virte M, Mirisola E, Sciamanna M, Panajotov K. Asymmetric dwell-time statistics of polarization chaos from free-running VCSEL. *Opt Lett* 2015; **40**: 1865–1868.

3 Virte M, Panajotov K, Thienpont H, Sciamanna M. Deterministic polarization chaos from a laser diode. *Nat Photon* 2013; **7**: 60–65.

4 Wolf A, Swift JB, Swinney HL, Vastano JA. Determining Lyapunov exponents from a time series. *Phys D* 1985; **16**: 285.

5 Virte M, Mercier E, Thienpont H, Panajotov K, Sciamanna M. Physical random bit generation from chaotic solitary laser diode. *Opt Express* 2014; **22**: 17271.

6 Kanter I, Frydman A, Ater A. Utilizing hidden Markov processes as a tool for experimental physics. *Eur Lett* 2005; **69**: 798.

7 Grassberger P, Procaccia I. Estimation of the Kolmogorov entropy from a chaotic signal. *Phys Rev A* 1983; **28**: 2591.

8 Grassberger P, Procaccia I. Characterization of strange attractors. *Phys Rev Lett* 1983; **50**: 346.

9 Fraedrich K, Wang R. Estimating the correlation dimension of an attractor from noisy and small datasets based on re-embedding. *Phys D* 1993; **65**: 373.

10 Virte M, Sciamanna M, Mercier E, Panajotov K. Bistability of time-periodic polarization dynamics in a free-running VCSEL. *Opt Express* 2014; **22**: 6772.
